# Revision Arthroplasty in the Haemophiliac Patient

**DOI:** 10.1155/2013/348080

**Published:** 2013-05-02

**Authors:** A. P. Molloy, B. J. O'Neill, L. Molloy, B. White, H. Smyth, T. Mc. Carthy

**Affiliations:** ^1^Department of Trauma and Orthopaedics, St. James's Hospital, Dublin, Ireland; ^2^National Centre for Inherited Coagulation Disorders, St. James's Hospital, Dublin, Ireland

## Abstract

Arthroplasty in the haemophiliac patient is associated with higher rates of infection and is traditionally performed in a younger age group. Despite this there is little evidence in the literature regarding revision arthroplasty in this cohort of patients. We describe the case of a periprosthetic fracture in a haemophiliac patient requiring revision arthroplasty, who did not consent to receiving blood products due to religious beliefs, with a successful outcome.

## 1. Introduction

We describe a revision arthroplasty for a periprosthetic fracture in a patient with severe Factor VIII deficiency.

## 2. Case Report

**Figure 1:**
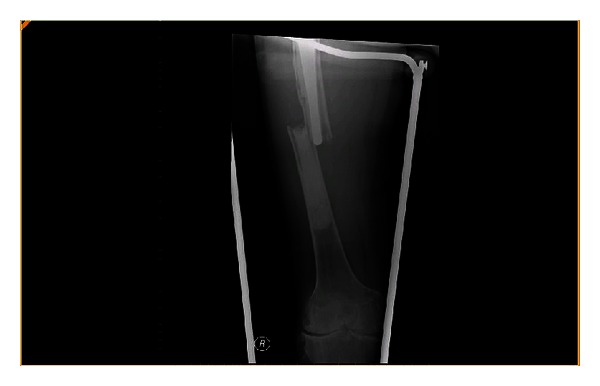
Periprosthetic fracture of right femoral prosthesis.

**Figure 2:**
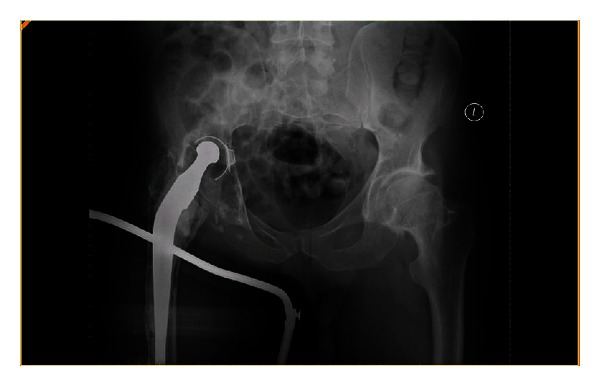
AP pelvis—periprosthetic fracture in Thomas splint demonstrating acetabular loosening.

A 46-year-old gentleman with a severe Factor VIII deficiency was referred from a tertiary referral centre with a periprosthetic fracture of the right femur (Vancouver B2) [1] (Figures 1 and 2). He had a primary hip replacement previously performed 25 years in another institution with subsequent revision 5 years later. His haemoglobin and platelets on admission were 14.1 g/dL and 232 × 10^9^/L, respectively, but he would not consent to receiving blood products during any procedures.

With a multidisciplinary team approach, involving the orthopaedic surgeons, the haematology department, and the medical social workers the risks of the procedure were explained to the patient along with the likelihood of necessitating blood transfusion given the planned operation.

Under the careful management of the haematologists, normovolemic haemodilution was carried out, with the patient given erythropoietin (EPO) pre-operatively, increasing the haemoglobin level to above 17 g/dL with autologous transfusion if necessary. The patient did agree that given the risks involved blood transfusion could be carried out as a “last resort.”

As per normal protocol for haematology arthroplasty the patient was given a preoperative bolus factor rise to 100% and a continuous perioperative ADVATE (Baxter) (anti-haemophilic factor (recombinant)/plasma/albumin free method) infusion with his preoperative haemoglobin measured at 17.4 g/dL.

The patient underwent revision arthroplasty with impaction bone grafting. A trochanteric slide was carried out, the acetabular cup removed, and a central defect exposed but with good anterior and posterior columns. Impaction bone grafting was performed using a human bone allograft frozen femoral head (CBB-08-03-2008), medial wall mesh (Howmedica), and a 52 mm cemented Exeter Contemporary flanged cup. 

**Figure 3:**
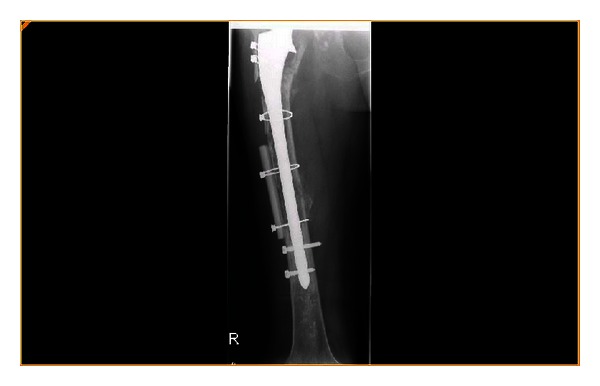
Postoperative fixation of periprosthetic fracture.

**Figure 4:**
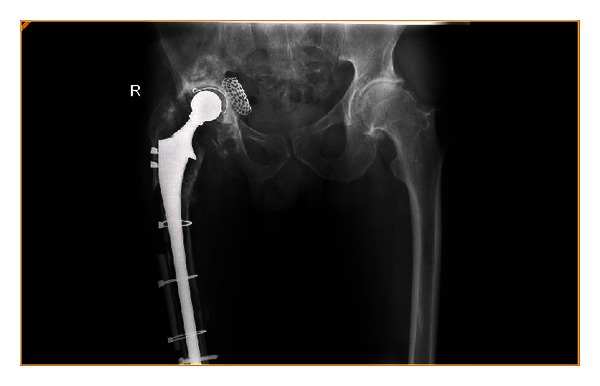
Postoperative fixation of periprosthetic fracture.

A trochanteric osteotomy was carried out to reveal an eggshell segmented femur. The previous cement was removed and a cemented 275 mm distal locking prosthesis was inserted (Howmedica) supplemented with a distal fibular allograft strut (Figures 3 and 4). Closure was uncomplicated and the patient transferred to the intensive care unit (ICU).

A continuous postoperative ADVATE (Baxter) infusion was given for a total of 9 days and discontinued on the advice of the haematologists, who were reviewing the patients daily. Haemoglobin levels decreased to a trough of 6.2 g/dL on the sixth postoperative day and as the patient was asymptomatic, he was treated with iron supplementation and did not require any blood transfusion. Levels gradually increase to 10.6 g/dL 2 weeks postoperatively.

The only noted complication postoperatively was a spike in temperature caused by blood culture which confirmed peripherally inserted central catheter (PICC) sepsis. This was treated by intravenous antibiotic therapy with no systemic sequelae. The patient was managed nonweight bearing for 6 weeks and discharged to a rehabilitation unit at 4 weeks postoperatively.

At the scheduled three-month follow-up, the patient was mobilising independently, was pain-free, and satisfied with his outcome. There was no evidence of infection and the patient will be monitored in the future at the joint haematology-orthopaedic clinic.

## 3. Discussion

End stage haemophiliac arthropathy is associated with impaired function and a significant decrease in the patients' quality of life [2]. However, arthroplasty in the haemophiliac patient is associated with higher risk of infection and carried out at a younger age than the normal population [3]. 

Revision surgery in the normal population increases the risk of both infection and dislocation substantially [4]. To carry out revision surgery in the haemophiliac patient secondary to a periprosthetic fracture increases these risks further. 

The role of the multidisciplinary team is very evident in the treatment of haemophiliac arthropathy [5]. By the nature of the disease, these patients are at risk of bleeding; thus revision arthroplasty is a significant risk for blood loss. Patients undergoing such surgery must be counselled, optimized, and educated to the significant risks involved. Surgery should not be rushed at the expense of a thorough preoperative workup. Our case was further complicated by the wishes of the patient, which should be adhered to as far as possible. The management of the haematological disorders is improving rapidly as is demonstrated by the lack of blood transfusion required, despite the background pathology.

Current literature reports an incidence of periprosthetic infection in the haemophiliac patient at up to 18% [6]. However these figures are reported after primary joint replacement with little evidence in the literature for revision arthroplasty. Given that these patients have numerous comorbidities including hepatitis and human immunodeficiency virus (HIV), we expect a higher rate for revision patients [6, 7].

This patient received close monitoring in the preoperative, perioperative, and immediate postoperative time periods. The patient was optimised prior to surgery and educated regarding the risks involved. The patient's wishes were respected and the multidisciplinary team involved ensured a successful outcome. However given the risks involved for the development of periprosthetic failure, all haemophiliac patients undergoing revision surgery require close monitoring for the next few years.

## References

[1] Duncan CP, Masri BA (1995). Fractures of the femur after hip replacement. *Instructional Course Lectures*.

[2] Schick M, Stucki G, Rodriguez M (1999). Haemophilic; arthropathy: assessment of quality of life after total knee arthroplasty. *Clinical Rheumatology*.

[3] Aledort LM, Haschmeyer RH, Pettersson H (1994). A longitudinal study of orthopaedic outcomes for severe factor-VIII-deficient haemophiliacs. *Journal of Internal Medicine*.

[4] Pulido L, Ghanem E, Joshi A, Purtill JJ, Parvizi J (2008). Periprosthetic joint infection: the incidence, timing, and predisposing factors. *Clinical Orthopaedics and Related Research*.

[5] Lobet S, Pendeville E, Dalzell R (2008). The role of physiotherapy after total knee arthroplasty in patients with haemophilia. *Haemophilia*.

[6] Hicks JL, Ribbans WJ, Buzzard B (2001). Infected joint replacements in HIV-positive patients with haemophilia. *Journal of Bone and Joint Surgery B*.

[7] Powell DL, Whitener CJ, Dye CE, Ballard JO, Shaffer ML, Eyster ME (2005). Knee and hip arthroplasty infection rates in persons with haemophilia: a 27 year single center experience during the HIV epidemic. *Haemophilia*.

